# Incompatibility of the short-acting benzodiazepine remimazolam with common perioperative medication

**DOI:** 10.1186/s12871-024-02613-7

**Published:** 2024-07-11

**Authors:** Pascal Hofmann, Lena Bachmann, Pia Brümmer, Berthold Drexler

**Affiliations:** 1 University Hospital Tübingen, Department of Anaesthesiology and Intensive Care Medicine, Tübingen, Germany; 2 Department of Anaesthesiology, Christchurch, New Zealand

**Keywords:** Coadministration, Incompatibility, Interaction, Precipitation, Remimazolam, Simulated y-site administration

## Abstract

**Background:**

Remimazolam is a relatively new benzodiazepine with growing use in procedural sedation and general anaesthesia. Initiated by case reports, the physical incompatibility of remimazolam with ringer’s acetated and ringer’s lactated solution has been reported. More recently, remifentanil, fentanyl, rocuronium, vecuronium, dexmedetomidine, and midazolam, have been investigated and suggested safe for coadministration with remimazolam. Apart from case reports, incompatibility for other frequently used drugs remains unknown.

**Methods:**

Sixty-five drugs and intravenous fluids were tested for possible precipitation with remimazolam in a simulated y-site administration. Equal volumes of the test drug were injected into the remimazolam solution, examined and photo documented at 1, 15, 30 and 60 min after mixture. Examination was taken by two independent investigators. pH was measured before, and 60 min after mixing the drugs.

**Results:**

Seventeen (26.15%) drugs or fluids showed precipitation, 47 (72.31%) did not show any sign of interaction. Propofol could not be assessed, because of the turbidity of the substance itself. Precipitation occurred immediately and remained stable in all timestamps. The incompatible drug-remimazolam-mixtures had a median pH of 7.15 (6.67, 8.01), the non-precipitating mixtures a median pH of 4.75 (3.8, 5.6). The pH-values of both groups were significantly different (Mann-Whitney-U-test; *p* < .00001). There is an increasing risk for precipitation with more basic baseline pH-levels of the tested drug. No interaction was seen in baseline pH below 5.

**Conclusions:**

Remimazolam (Byfavo^®^) is incompatible with ampicillin/ sulbactam, calcium gluconate, clindamycin, dexamethasone, dimenhydrinate, an 148mval/l electrolyte - glucose 1% solution (E148G1^®^), furosemide, a 4% gelatine volume expander (gelafundin^®^^)^, heparin sodium, insulin, meropenem, sodium bicarbonate 8.4%, prednisolone, the crystalloid infusions jonosteril^®^ and sterofundin^®^, thiopental and tranexamic acid. The results strongly affirm remimazolam’s safety requirements: A separate line for remimazolam and an approved compatible baseline infusion is mandatory and an alternative way to administer bolus medication is required.

## Background

Remimazolam is a relatively new, ultra-short-acting benzodiazepine [1]. It is approved in the US, EU, UK, EEA, China and South Korea for procedural sedation and in Japan and South Korea for general anaesthesia. The extension of its use and broader approval for general anaesthesia is to be expected [2–6].

Remimazolam can be administered as boluses for procedural sedation or as a continuous infusion for general anaesthesia [3, 5, 7, 8]. So far, according to the product information sheet, safe co-administration has only been approved for glucose (5%) solution for injection, glucose (20%) solution for injection, glucose (5%) with sodium chloride (0.45%) solution for injection, Ringer’s Solution and sodium chloride (0.9%) solution for injection. Because of missing further data, the information sheet states that the commercially available formulation of remimazolam, Byfavo^®^ (PAION, Germany) must not be mixed or co-administered through the same infusion line with other medicinal products [7]. 

Because of this, a separate iv line for the administration of remimazolam is obligatory, especially when it is administered continuously, and any intended or accidental co-administration might bear unforeseeable risks. An incompatibility with Ringer’s lactate and Ringer’s acetate Solution has been reported by Sasaki et al. in 2021 [9], leading to a complete occlusion of the used iv line during co-administration. Sung et al. presented two case reports describing similar incompatibility with plasma-lyte 148 solution [10]. Decreased solubility of remimazolam in pH > 4 was assumed as one of the possible reasons for the precipitation [9]. This pH-related reduced solubility and the incompatibility with lactated, acetated and bicarbonated Ringer’s Solution is now also described in the Byfavo^®^ product information sheet [7]. There are many known incompatibility reactions forming precipitations, for example thiopental and muscle relaxants [11], furosemide and midazolam [12], vancomycin and piperacillin/tazobactam [13], sugammadex and protamine [14], piritramide and cefazolin [15] or ceftriaxone and theodrenaline/cafedrine [16].

Kondo et al. recently claim to have shown that there is no physical interaction between remimazolam and remifentanil, fentanyl, rocuronium, vecuronium, dexmedetomidine, and midazolam, concluding compatibility from the absence of detectable precipitation [17].

Physical or chemical drug-drug interactions can lead to formation of precipitations, which could block lines and result in loss of intravenous access, deposit in vessels or embolise and accumulate in organs [18–20]. The absence of visible changes does however not safely exclude drug-drug interaction, which could lead in a loss of the intended effects or even result in gaining unwanted and unknown side effects.

Further investigation about remimazolam is needed to identify the risks of co-administration, to avoid possible complications and provide a safe handling.

## Methods

Sixty-five drugs and intravenous fluids that are commonly used in perioperative medicine in Europe were identified to be tested for possible precipitation with remimazolam. The drugs were prepared in the highest common concentration in clinical use and according to each drug’s data and information sheet. Remimazolam was prepared in a concentration of 2.5 mg/ml. Drugs that were reconstituted from powder were drawn up by using a 5 μm Mini-Spike^®^-filter (B. Braun, Melsungen, Germany) to avoid the presence of remaining particles in the test solution. All drugs were drawn up in plastic syringes (B. Braun, Melsungen, Germany), according to hospital standards. Equal volumes of remimazolam and the test drug, respectively, were combined by injecting the test drugs into a remimazolam prefilled transparent tube (Nunc^®^ cell culture tubes, Fisher Scientific, Germany) to simulate a y-site administration. Remimazolam was injected first, then the test drug was added.

Two independent investigators (L.B. and B.D.) visually evaluated the formation of precipitation at 1, 15, 30 and 60 min after injection. At each of these timestamps, a photo of the test vial, along with a remimazolam filled control vial, was taken in a comparable setup. (Nikon D90 on a tripod in front of a black background). Artificial room lighting with darkened windows was used for both visual examination and photo taking to avoid interference by changing daylight illumination during the trial. All experiments were performed at room temperature and the temperature was recorded at the time of injection. Any visible turbidity of the mixed solution in comparison to the remimazolam solution was recorded as precipitation. A specification could be made by additional comments. All findings were recorded on a case report form.

The pH values of the test drugs were measured with a pH-meter (761 Calimatic Knick, Germany with IDS Electrode BlueLine 14 pH IDS, SI Analytics, Germany) before and 60 min after mixing them with remimazolam. The pH-meter was calibrated with Hamilton DuraCal liquid pH buffer solutions pH 4.01, pH 7.0 and pH 9.21 immediately before starting the experiment. Due to technical limitations, only test drugs with a vial volume of more than 1 ml could be measured. Baseline pH levels of 14 drugs were taken at a later time by the same investigators, with the same equipment and according to the same protocol.

Microsoft Excel 365^®^ was used for descriptive statistics. Mann-Whitney U Test Calculator was used for statistical testing [21]. pH-data is given as median with 25% and 75% quartiles.

Remimazolam (Byfavo^®^) was kindly provided by PAION, Germany.

## Results

65 drugs and intravenous fluids were tested according to the described methods. The room temperature was between 20.7 °C and 21.7 °C. seventeen (26.15%) tested drugs showed precipitation in combination with remimazolam, forty-seven (72.31%) combinations did not show a sign of interaction, turbidity, or precipitation (Table 1). Propofol could not be assessed, because of the turbidity of the substance itself.

When precipitation was found, it occurred immediately in the first inspection at 1 min and remained present, without future dissolution until the last inspection at 60 min. Only for clindamycin, precipitation was found at 1 and 15 min after combination, no precipitation was found at 30 min and a minimal turbidity described at 60 min.

**Table 1:** Drug or intravenous fluid, manufacturer, concentration, pH of the drug, pH of the mixture and the precipitation status. Values indicated (‡) have been measured at a separate time

Active substance	Manufacturer	Concentration	pH active substance	pH mixture	Precipitation
Remimazolam besylate	Paion	2.5 mg/ml	3.44		n.a.
Propofol	Fresenius Kabi Deutschland GmbH	10 mg/ml	8.10	3.36	n.a.
Ampicillin/ sulbactam	Pfizer Pharma GmbH	30 mg/ml	8.67	8.32	Positive
Calcium gluconate	B. Braun Melsungen AG	1mmol/ml	6.67	5.26	Positive
Clindamycin	Chephasaar Chemisch-Pharmazeutische Fabrik GmbH	150 mg/ml	6.77	6.85	Positive
Dexamethasone	Merck	4 mg/ml	8.01^‡^	6.62	Positive
Dimenhydrinate	Klinge Pharma GmbH	6.25 mg/ml	7.26	5.60	Positive
E148G1^®^	Serumwerk Bernburg AG	Pure	5.52	5.02	Positive
Furosemide	Ratiopharm GmbH	10 mg/ml	8.94^‡^	5.55	Positive
Gelafundin^®^	B. Braun Melsungen AG	40 mg/ml	7.15	5.68	Positive
Heparin sodium	LEO Pharma GmbH	5.000 IE/ml	6.64	5.83	Positive
Insulin	Eli Lilly GmbH	100 IE/ml	7.19	4.19	Positive
Jonosteril^®^	Fresenius Kabi Deutschland GmbH	Pure	6.15	5.28	Positive
Meropenem	Fresenius Kabi Deutschland GmbH	20 mg/ml	7.72	7.72	Positive
Sodium bicarbonate 8.4%	Fresenius Kabi Deutschland GmbH	Pure	8.03	8.15	Positive
Prednisolone	Dermapharm GmbH	50 mg/ml	6.69	6.37	Positive
Sterofundin^®^	B. Braun Melsungen AG	Pure	5.25	4.90	Positive
Thiopental	Inresa Arzneimittel GmbH	2.5 mg/ml	10.81	10.01	Positive
Tranexamic acid	Carinopharm GmbH	100 mg/ml	7.00	6.29	Positive
Adrenaline	Infectopharm Arzneimittel und Consilium GmbH	1 mg/ml	3.79	3.60	Negative
Cafedrine/ theodrenaline	Ratiopharm GmbH	100&5 mg/ml	5.02^‡^	4.92	Negative
Amiodarone	Hameln Pharma GmbH	50 mg/ml	3.88	3.25	Negative
Aqua	Fresenius Kabi Deutschland GmbH	Pure	7.54	3.63	Negative
Argipressin	Orpha-Devel Handels und Vertriebs GmbH	20IE/ml	3.84^‡^	3.60	Negative
Atropine	B. Braun Melsungen AG	0.5 mg/ml	3.32^‡^	3.58	Negative
Butylscopolamine	Sanofi	20 mg/ml	4.10^‡^	3.59	Negative
Cefazoline	Fresenius Kabi Deutschland GmbH	40 mg/ml	5.61	4.22	Negative
Cefuroxime	MIP Pharma GmbH	30 mg/ml	7.33	4.43	Negative
Cimetidine	Ratiopharm GmbH	100 mg/ml	5.47^‡^	5.65	Negative
Clonidine	Boehringer Ingelheim	150 µg/ml		3.60	Negative
Dimetindene maleate	Pharmore GmbH	4 mg/ml	4.20	3.79	Negative
Dobutamine	Carinopharm GmbH	5 mg/ml	3.77	3.65	Negative
Esmolol	Orpha-Devel Handels und Vertriebs GmbH	10 mg/ml	5.02	4.81	Negative
Fentanyl	Rotexmedica GmbH Arzneimittelwerk	78.5 µg/ml	4.96	3.66	Negative
Fluconazole	Fresenius Kabi Deutschland GmbH	2 mg/ml	5.39	3.64	Negative
Flumazenil	Fresenius Kabi Deutschland GmbH	0.1 mg/ml	4.19	3.68	Negative
Glucose 40%	Fresenius Kabi Deutschland GmbH	400 mg/ml	3.37	3.40	Negative
Glucose 5%	Fresenius Kabi Deutschland GmbH	50 mg/ml	4.53	3.60	Negative
Potassium chloride	Fresenius Kabi Deutschland GmbH	1mmol/ml	5.72	3.68	Negative
Esketamine	Pfizer Pharma GmbH	25 mg/ml		3.50	Negative
Lidocaine	Aspen Pharma Trading Limited	20 mg/ml	6.58	4.61	Negative
Magnesium sulfate	Inresa Arzneimittel GmbH	100 mg/ml	6.50	3.90	Negative
Mannitol	Serag-Wiessner GmbH & Co. KG	150 mg/ml	5.85	3.58	Negative
Metoclopramide	Ratiopharm GmbH	5 mg/ml	6.22^‡^	3.61	Negative
Metamizole	Ratiopharm GmbH	1 g/ml		6.31	Negative
Metoprolol	Recordati Pharma GmbH	1 mg/ml	5.93	3.90	Negative
Metronidazole	B. Braun Melsungen AG	5 mg/ml	5.53	4.31	Negative
Midazolam	Hameln Pharma GmbH	1 mg/ml	3.31^‡^	3.41	Negative
Milrinone	Carinopharm GmbH	1 mg/ml	3.80	3.77	Negative
Sodium chloride 0.9%	B. Braun Melsungen AG	9 mg/ml	5.43	3.62	Negative
Naloxone	Hameln Pharma GmbH	0.4 mg/ml	3.45^‡^	3.50	Negative
Neostigmine	Carinopharm GmbH	0.5 mg/ml	5.28^‡^	3.70	Negative
Glycerol trinitrate	Pohl-Boskamp	1 mg/ml	3.68	3.49	Negative
Noradrenaline	Sanofi	1 mg/ml	4.27	3.59	Negative
Ondansetron	Carinopharm GmbH	2 mg/ml	3.53^‡^	3.50	Negative
Paracetamol	B. Braun Melsungen AG	10 mg/ml	5.09	4.12	Negative
Piperacillin/ tazobactam	Fresenius Kabi Deutschland GmbH	90 mg/ml	5.07	4.60	Negative
Piritramide	Hameln Pharma GmbH	7.5 mg/ml	3.91^‡^	3.90	Negative
Protamine	LEO Pharma GmbH	1400IE/ml	6.48	3.81	Negative
Remifentanil	Fresenius Kabi Deutschland GmbH	100 µg/ml	3.08	3.21	Negative
Rocuronium	Inresa Arzneimittel GmbH	10 mg/ml	4.06	4.05	Negative
Succinylcholine	Takeda	50 mg/1 ml	3.17^‡^	3.19	Negative
Sufentanyl	Piramal Critical Care B.V.	5 µg/ml	5.60	3.66	Negative
Urapidil	CARINOPHARM GmbH	5 mg/ml	5.82	3.84	Negative
Vancomycin	Hospira Deutschland GmbH	50 mg/ml	3.28	3.40	Negative
Vecuronium	Inresa Arzneimittel GmbH	1 mg/ml	3.99	3.86	Negative

Remimazolam had a baseline pH of 3.44 in a concentration of 2.5 mg/ml. Because of technical limitations with small test-volumes, the baseline pH of 17 drugs could not be taken in the initial experiment. Therefore, the baseline pH of 14 of these drugs was measured in the same laboratory-setup by the same investigators and with the same equipment at a later stage. Three drugs, esketamine, metamizole and clonidine, were not followed up in that way, because hospital supplies had changed to different brands in the meantime.

The 17 drugs interacting with remimazolam and forming precipitations had a median pH of 7.15 (6.67, 8.01) with a minimum of 5.25 for sterofundin^®^ infusion and a maximum of 10.81 for thiopental. The mixed solutions had a median pH of 5.83 (5.28, 6.85) with a minimum of 4.19 for insulin with remimazolam and a maximum with 10.01 for thiopental with remimazolam.

The median pH difference between remimazolam and the mixtures was + 2.39 (+ 1.84, + 3.41) with a minimum of + 0.75 for insulin and a maximum of + 6.57 for thiopental in the precipitation forming group.

The drugs not showing precipitation had a median pH of 4.75 (3.8, 5.6) with a minimum of 3.08 for remifentanil and a maximum of 7.54 for aqua ad injectabila. The mixtures had a median pH of 3.66 (3.59, 3.9) with a minimum of 3.19 for suxamethonium chloride and a maximum of 6.31 for metamizole.

The median pH difference between remimazolam and the mixtures was + 0.22 (+ 0.15, + 0.46) with a minimum of -0.25 for suxamethonium chloride and a maximum of + 2.87 for metamizole.

**Fig. 1:**
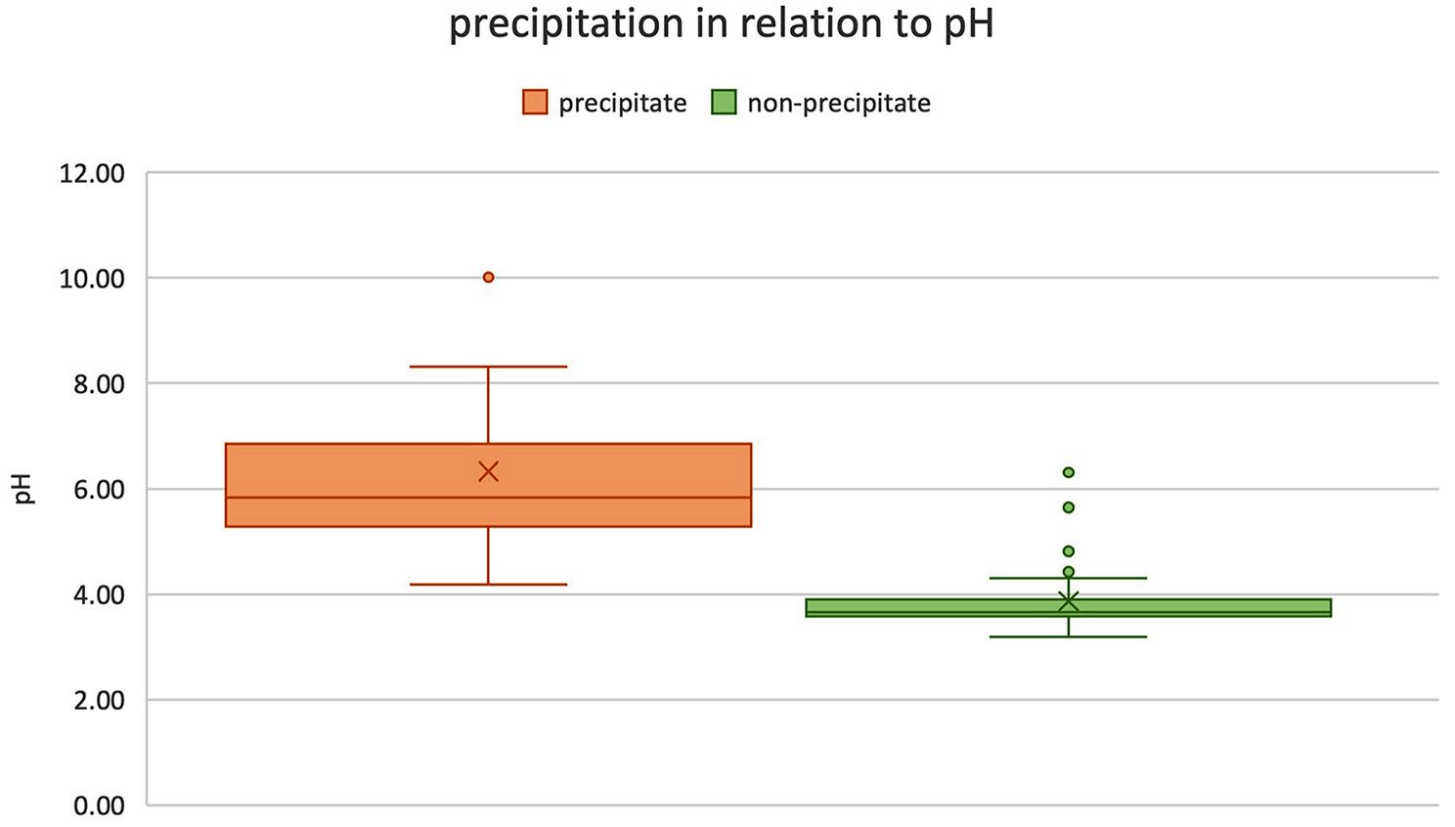
pH level of the drug-remimazolam mixtures in relation to the precipitation status. Box and whisker chart showing pH levels of the precipitating mixtures on the left (red) and the non-precipitating mixtures on the right (green). The box shows median and Quartiles, the whiskers are defined by 1.5 times interquartile range. Outliers are shown as dots. Mean pH is indicated as x. The baseline pH of pure remimazolam is, 3.44.

Figure 1 indicates a correlation between higher pH levels and the formation of precipitations. Statistically comparing the two samples by testing with a Mann-Whitney-U-test, two-tailed, shows a U-value of 25, Z-score of -5.68502 and a p-value of < 0.00001. Therefore, a significant pH difference between the groups is confirmed at *p* < .05

**Fig. 2:**
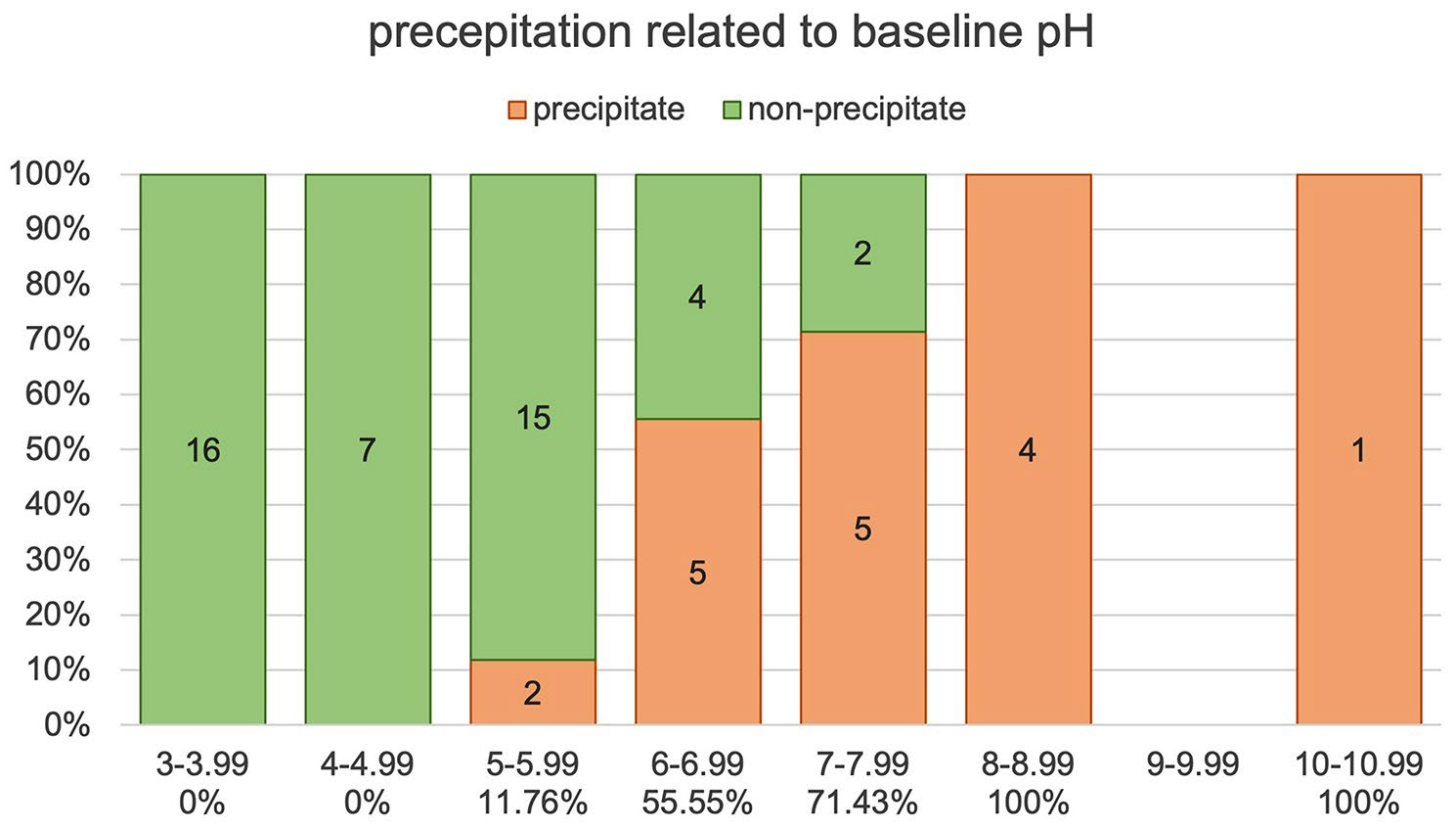
pH of the test drugs before mixing, grouped in pH ranges on the x axis. Fractions of precipitating drugs in red and non-precipitating drugs in green presented on the y axis. The absolute numbers shown in each respective column. The fraction of precipitating drugs in each individual column is also shown as percentage under each column. There was no drug tested with a baseline pH between 9 and 9.99

Figure 2 shows the baseline pH level in pH groups. Below a pH of 5, fraction of precipitation is 0%, whereas above 8, 100% of the tested drugs precipitate. Between baseline pH 5 and 8, precipitation increases continuously with increasing pH of the substance mixed with remimazolam.

**Fig. 3:**
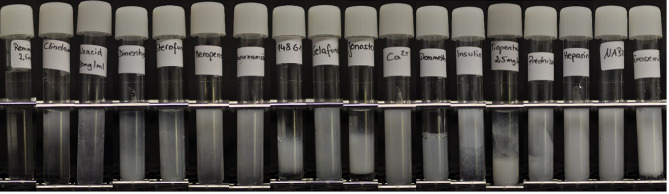
Precipitating mixtures at 1 min after injection. Tubes from left to right: remimazolam (plain), clindamycin, ampicillin/sulbactam, dimenhydrinate, sterofundin^®^, meropenem, tranexamic acid, E148G1^®^, gelafundin^®^, jonosteril^®^, calcium gluconate, dexamethasone, insulin, thiopental, prednisolone, heparin sodium, sodium bicarbonate, and furosemide

Precipitations showed different intensities, ranging from the formation of streaks and a slight turbidity to clumping of bigger particles and sedimentation (Fig. 3). However, a link between the intensity of the reaction and the pH of the mixture could not be observed.

## Discussion

The current study investigates the incompatibility of remimazolam (Byfavo^®^) with other commonly used drugs in anaesthesia and intensive care. The incompatibilities revealed by the current study are important to consider for safe drug administration.

About one fourth (26.15%) of the tested drugs and intravenous fluids showed physical interaction with remimazolam, leading to precipitation. The group of drugs or fluids that are incompatible is heterogeneous, containing crystalloid infusions, antibiotics, and various drugs. The onset of the precipitation is immediate, as shown in the early formation right after mixture and documented one minute after mixing the substances. The stability of the compound seems permanent, as no dissolvement of the precipitation could be observed, except for clindamycin. This study, however, cannot give information whether dilution with a large volume would result in dissolving of the precipitate.

The pH-change of remimazolam-drug mixture seems to be a predictive indicator for incompatibility. The precipitate groups pH levels are significantly more alkaline than the non-precipitate group. Conclusively, the less acidic the mixture is, the higher the risk for incompatibility (Fig. 2). However, a clear cut off in pH, from which the incompatibility starts, cannot be found. Also, pH cannot be the single reason for precipitation, as there is an overlap in pH levels from both groups.

The findings support the safety information sheet’s statement that solubility reduces at a pH of 4 and above, however, no precipitation has been found under the pH of 5. The results clearly show an increasing fraction for precipitation with increasing pH of the drug mixed with remimazolam, starting at a pH above 5.

Sasaki et al. [9] reported a relevant patient safety issue by the iv cannula occlusion while having remimazolam co-administered with ringer’s acetate, due to the slow but constant formation of particles. If change in pH is the main contributing factor to these formations, it seems reasonable to speculate that precipitation of remimazolam with venous blood could be a risk, as the average mixed venous blood pH is 7.36 [22]. This is close to the median pH of 7.15 of precipitation forming drugs in this study. Further investigation to clarify possible precipitation of remimazolam with venous blood would be suggested as the constant infusion of remimazolam into venous blood could lead to a constant formation of particles, like the case presented by Sasaki et al.

A limitation of this study is the visual inspection without further testing by checking for Tyndall effect, measure turbidity or perform spectrometric analysis. This would be required for further investigation. Henceforth, observed precipitation can clearly be described as incompatible, whereas non-observed turbidity could have underlain but not by eyesight observable interactions. The absence of precipitation henceforth, cannot be considered sufficient proof of compatibility.

Baseline pH data is missing for 3 drugs (4.6%), for 14 drugs the baseline pH was taken at a later stage, the standardised setup as well as equipment and investigator consistency should minimise possible errors associated with this.

Our study describes precipitation but cannot further elaborate which physical or chemical reaction takes place, nor which component of each drug, the active substance, or an adjuvant, is reacting. There is a high chance that drug formulations of the same active substance but provided by different supplier, will show the similar compatibilities or incompatibilities. However, this study is limited to the specific brands investigated.

Remimazolam and the test drugs were mixed directly in a tube, which might not reflect the real-life situation in clinical practice, where drugs are normally diluted and administered at different ports into a baseline infusion. This might limit transferability of the experimental findings into daily clinical practice. Furthermore, Sasaki et al. [9] showed that lower concentrations of remimazolam and higher infusion rates reduce precipitation, so that precipitation would not happen as obvious as in the experimental setting.

Clinically, precipitation can mean a loss of the desired effect, an undesired side effect, sedimentation and occlusion in lines and cannulas, possible vein irritation, systemic distribution of particles with possible capillary occlusions or even pulmonary embolism. Using the incompatible infusion solutions, as well as administering boluses or infusions into the same iv line as remimazolam must be carefully avoided.

## Conclusion

Remimazolam (Byfavo^®^) is incompatible with the commonly used peri-operative medications ampicillin/sulbactam, calcium gluconate, clindamycin, dexamethasone, dimenhydrinate, the 148mval/l electrolyte - glucose 1% solution E148G1^®^, furosemide, the 4% gelatine volume expander gelafundin^®^, heparin sodium, insulin, the commonly used crystalloids jonosteril^®^ and sterofundin^®^, meropenem, sodium bicarbonate 8.4%, prednisolone, thiopental and tranexamic acid, although, as a matter of principle, this applies of course only to the specific galenic formulation given by the pharmaceutic manufacturer and tested in our study. Administration of remimazolam (Byfavo^®^) and one of the above-mentioned drugs over the same iv line must be avoided. The results strongly affirm remimazolam’s safety requirements: A separate line for remimazolam and an approved compatible baseline infusion is mandatory and an alternative way to administer bolus medication is required.

## Data Availability

All data generated during the current study, including report forms and photo documentation, are available from the corresponding author on reasonable request.
